# *Eucalyptus camaldulensis* Dehnh Leaf Essential Oil from Palestine Exhibits Antimicrobial and Antioxidant Activity but No Effect on Porcine Pancreatic Lipase and α-Amylase

**DOI:** 10.3390/plants12223805

**Published:** 2023-11-08

**Authors:** Nidal Jaradat, Nawaf Al-Maharik, Mohammed Hawash, Mohammad Qadi, Linda Issa, Rashad Anaya, Ayham Daraghmeh, Lobna Hijleh, Tasneem Daraghmeh, Amal Alyat, Ro’a Aboturabi

**Affiliations:** 1 Department of Pharmacy, Faculty of Medicine and Health Sciences, An-Najah National University, Nablus P.O. Box 7, Palestine; mohawash@najah.edu (M.H.); l.issa@najah.edu (L.I.); rashadanaya28@gmail.com (R.A.); ayhamabubshara@gmail.com (A.D.); lobna.hijli@gmail.com (L.H.); tasnimdaraghma@gmail.com (T.D.); amaalaam112233@gmail.com (A.A.); roro.abutrabi@gmail.com (R.A.); 2 Department of Chemistry, Faculty of Sciences, An-Najah National University, Nablus P.O. Box 7, Palestine; 3 Department of Biomedical Sciences, Faculty of Medicine and Health Sciences, An-Najah National University, Nablus P.O. Box 7, Palestine; m.qadi@najah.edu

**Keywords:** *Eucalyptus camaldulensis*, essential oil, GC-MS, antioxidant, antilipase, antiamylase, antimicrobial

## Abstract

*Eucalyptus camaldulensis* Dehnh is a tree species that is commonly used for various purposes, including forestry, agroforestry, and conservation. The present investigation was designed to determine the composition of *E. camaldulensis* leaves essential oil and estimate its free radicals, porcine pancreatic lipase, α-amylase inhibitory, and antimicrobial properties in vitro. The chemical constituents were analyzed using the gas chromatography-mass spectrometry (GC-MS) technique. DPPH (2,2-diphenyl-1-picrylhydrazyl), p-nitrophenyl butyrate, and 3,5-dinitro salicylic acid (DNSA) methods were employed to estimate the antioxidant, antiobesity, and antidiabetic effects of the essential oil. The microdilution assay was employed to assess the antimicrobial efficacy of the substance against a total of seven distinct microbial species. The GC-MS results revealed that *E. camaldulensis* essential oil contains 52 components that makeup 100% of the entire oil. The main chemical constituents in *E. camaldulensis* essential oil are *p*-cymene (38.64%), followed by aromadendrene (29.65%), and 1,8-cineol (6.45%), with monocyclic monoterpene being the most abundant phytochemical group, followed by the sesquiterpene hydrocarbon group, representing 44.27 and 31.46%, respectively. The essential oil showed a weak antioxidant effect and had no antilipase or antiamylase effects. At the same time, the oil showed a strong antimicrobial effect against methicillin-resistant *Staphylococcus aureus* (MRSA), *Staphylococcus aureus*, and *Proteus vulgaris*, which was even more potent than the positive controls, ciprofloxacin and ampicillin, which had MIC doses of 0.2 ± 0.01, 0.2 ± 0.01, and 6.25 ± 0.1 µg/mL, respectively. It also has a strong anti-*Candida albicans* effect with a MIC of 0.2 ± 0.01 µg/mL. In light of these findings, in vivo studies should be conducted to determine the efficiency of the *E. camaldulensis* essential oil in treating microbial infections.

## 1. Introduction

Essential oils have piqued the interest of many industries, including aromatherapy, cosmetics, pharmaceuticals, and food preservation [1,2]. Essential oils are a remarkable class of bioactive compounds that occur naturally. The bioactive compounds found in essential oils impart distinctive aromas and medicinal properties [3]. The capacity of Essential oils to operate as powerful antibacterial, antifungal, and antioxidant agents has positioned them as important alternatives for synthetic chemicals in food preservation, aligning with the global shift toward more eco-friendly, healthier, and sustainable consumption practices [4].

*Eucalyptus camaldulensis* Dehn., often known as River Red Gum, is a perennial evergreen tree that falls under the genus Eucalyptus within the family Myrtaceae. The Eucalyptus genus encompasses more than 800 species, with its origins traced back to Australia and Tasmania. Over time, it has spread over the globe, owing to its remarkable adaptability and rapid growth. This successful introduction of Eucalyptus can be attributed to its ability to easily acclimate to different environments and its capacity for swift development [5].

*E. camaldulensis* (formerly *Eucalyptus rostrata* Schl.) is one of the most broadly distributed *Eucalyptus* species. Additionally, it is widely acknowledged as one of the most extensively cultivated tree species globally, with the potential to attain a lifespan ranging from 500 to 1000 years. *E. camaldulensis leaves* and their essential oil have been utilized for centuries as Aboriginal traditional remedies due to their antipyretic, anti-inflammatory, and antibacterial properties [5,6]. The *E. camaldulensis* plant was utilized by the Indigenous population of Australia for medicinal purposes, encompassing a diverse range of ailments. These included digestive tract disorders such as colic, diarrhea, and dysentery, respiratory system afflictions like colds, coughs, asthma, pharyngitis, sore throat, and trachalgia, as well as the alleviation of pain stemming from open wounds and cuts, along with the management of muscle and joint pain [5,6].

Recent years have seen a significant increase in the study of *E. camaldulensis* as scientists attempt to verify the plant’s purported traditional therapeutic capabilities in the treatment of a diverse array of illnesses. Its essential oils are reported to be astringent, antiseptic, and anesthetic [7]. Moreover, a decoction of the leaves reportedly treats sore throat and various urinary and respiratory tract bacterial infections [8]. *E. camaldulensis* essential oil has shown promise as a skin care product, and its components have found their way into the food, drug, and beauty sectors as flavoring agents [9].

The recorded yields of essential oil obtained from *E. camaldulensis* leaves demonstrate notable variability, spanning from 0.5% to 2.53% [10]. These figures are comparatively lower than the reported essential oil yields for other species of Eucalyptus, which range from 1.12% to 3.0% [10]. Based on existing research, it has been observed that *E. camaldulensis* essential oil has a significant proportion of oxygenated monoterpenes (40.8–87.42%) and monoterpene hydrocarbons (5.7–52.2%) [10]. Conversely, the presence of oxygenated sesquiterpenes (4.9–39.6%) and sesquiterpene hydrocarbons (1.8–3.6%) is comparatively lower [10]. Variation in *E. camaldulensis* essential oil yields and chemical composition may result from a number of factors, including but not limited to plant genetic and/or epigenetic factors (natural and induced); nutrients of different soils; species’ acclimation to the Australian environment; ecotype differences; and environmental influences [10]. Variations in the chemical composition of *E. camaledulensis* essential oils have been observed by distinct seasonal patterns. Nevertheless, the eucalyptus essential oil constantly exhibits the presence of key components such as 1,8-cineole, *p*-cymene, *γ*-terpinene, and *β*-pinene. The chemical composition of *E. camaldulensis* EO allows for the division into two distinct categories: essential oil with a high concentration of 1,8-cineole, which makes up between 80 and 90% of its composition along with pinene, and essential oil with a low concentration of cineole, which contains significantly less 1,8-cineole [10].

As part of our team’s ongoing work on the search for potential drugs from plants, herein, we report the phytochemical profile of *E. camaldulensis* leaves essential oil from Palestine and estimate its free radicals, porcine pancreatic lipase, α-amylase inhibitory, and antimicrobial properties in vitro.

## 2. Results and Discussion

### 2.1. Phytochemistry

**Figure 1:**
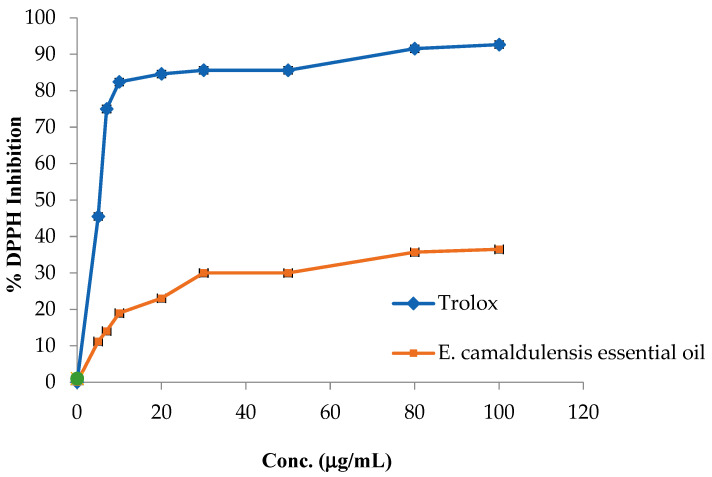
DPPH free radical suppressant effect by *E. camaldulensis* EO and Trolox.

The chemical makeup of the essential oil derived from *E. camaldulensis* was examined using gas chromatography-mass spectrometry (GC-MS), as depicted in Figure 1. This analytical technique facilitated the identification and quantification of a total of 52 components, collectively accounting for 100% of the oil. Table 1 presents the names, retention times, retention indices (RI), and percentages of detected constituents for the essential oil of *E. camaldulensis*. The hydrodistillation process of *E. camaldulensis* leaves led to a 1.11% yield of light yellow essential oil. This yield is greater than the yield obtained from *E. camaldulensis* leaves collected in Pakistan and the Morocco region, which ranged from 0.90% to 0.98% [11,12]. In their study, Moudachirou et al. [13] documented the oil concentration found in several places around Benin, which ranged from 0.6% to 1.4%. The variation in climatic conditions is contingent upon the time of year. The highest yield of essential oil was achieved from *E. camaldulensis* trees harvested in Taiwan, with a range of 2.3% to 3.0%. The essential oil yields of various Eucalyptus species exhibit a range of 1.2% to 3% (*w*/*w*) [10].

*ο*-Cymene (38.64%), aromadendrene (29.65%), and 1,8-cineole (6.45%) are the primary chemical components of *E. camaldulensis* essential oil. Furthermore, monocyclic monoterpene was the most common phytochemical group in the essential oil, accounting for 44.27%, followed by the sesquiterpene hydrocarbon group, which accounted for 31.46%. The studied essential oil is a cineole-poor oil.

Dellacasa et al. reported that the chemical compositions of the *E. camaldulensis* essential oil from Uruguay were *p*-cymene (30.8%), followed by spathulenol (15.3%), cryptone (12.5%), and terpinen-4-o1 (4.1%) [14].

*p*-Cymene, cyptone, and spathulenol with 22.9%, 14.1%, and 16.5%, respectively, were found to be the abundant compounds of *E. camaldulensis* essential oil from Australia [15]. Moreover, Barra et al. discovered 37 molecules in *E. camaldulensis* essential oil from Italy, which accounted for approximately 97.7% of the total essential oil, and its main constituents were *p*-cymene (27.8–42.7%), 1,8-cineole (4.1–39.5%), α-phellandrene (3.9–23.8%), spathulenol (2.1–15.5%), and cryptone (3.2–10.2%) [16]. Cheng et al. reported the identification of 20 compounds amounting to 97.58% in the *E. camaldulensis* leaf, of α-pinene (22.52%), *p*-cymene (21.69%), α-phellandrene (20.08%), 1,8-cineole (9.48%), γ-terpinene (9.36%), and limonene (4.56%) [17]. Ez-Ezzriouli reported the identification of 15 compounds representing 100% of the essential oil of E. camaldulensis, of which *p*-cymene (35.11%), γ-eudesmol (11.9%), L-linalool (11.51%), and piperitone (10.28%) were the major components [18].

The chemical constituents of *E. camaldulensis* essential oils have been documented in various regions, with 1,8-cineole reported as a main component with varying concentrations but within the same range. The 1,8-cineole content of essential oil was as follows: Greece (25.3–44.2%), Pakistan (34.4–40.0%) [11], Nigeria (32.8–70.4%) [19], and Taiwan (34.0–68.2%) [20]. The chemical components of Egyptian *E. camaldulensis* essential oil were determined by Abo Elgat et al., who found that spathulenol made up 20.84% of the oil, followed by *p*-cymene (15.16%), 1,8-cineole (12.01%), and sabinene (9.73%) [15].

According to the literature, 1,8-cineole, β-pinene, γ-terpinene, *p*-cymene, trans-pinocarveol, and terpinen-4-ol predominate in *E. camaldulensis* essential oils [21]. The essential oil of the *Eucalyptus camaldulensis* plant sourced from Palestine was found to be mostly composed of *p*-cymene hydrocarbon monoterpenes. Additionally, the oxygenated monoterpenoid 1,8-cineole was identified as the third most abundant compound in the oil.

There are several possible explanations for the distinctions in chemical composition between *E. camaldulensis* essential oils. These reasons can be divided into five major categories. First, the modification of plant genes through generations and hybridizations, both natural and artificial, may result in the production of volatile oils distinct from those found in various habitats. Furthermore, the variable composition of nutrients found in different soils and their subsequent accumulation in plant leaves can have a significant impact on plant metabolism. This, in turn, can lead to the synthesis of various bioproducts and essential oils comprising a range of chemicals in varying proportions. Furthermore, it is important to consider that the adaptation of the species to the Australian environment, where it has historically thrived, may exhibit variations when compared to trees that have been introduced and/or cultivated on plantations elsewhere. In addition, variations may emerge as a result of distinct ecotypes of *E. camaldulensis*. Differences may also arise due to variances in the plant component utilized for essential oil extraction and its developmental stage. Moreover, the essential composition differs when the plant material is dried or used fresh. Comprehending these parameters is vital for optimizing the production of essential oils through the cultivation of *E. camaldulensis* in suitable regions and under certain environmental circumstances [22].

### 2.2. Evaluation of the Antioxidant, Antilipase, and Anti-α-Amylase Effects

The DPPH assay was used to assess the antioxidant potential of *E. camaldulensis* essential oil. The DPPH (2,2-diphenyl-1-picrylhydrazyl) assay is a widely used method for assessing the antioxidant activity of various natural and synthetic compounds because it is simple and easy to perform, sensitive, no need for pre-knowledge, has good correlation with in vivo tests, can be adapted for high-throughput screening, and it can be used to assess the stability of antioxidants under different storage conditions [23].

Figure 1 shows that *E. camaldulensis* essential oil has weak antioxidant activity with an IC_50_ value of 398 ± 0.21 µg/mL compared with the positive control Trolox, which has a potent antioxidant effect with an IC_50_ dose of 5.01 ± 0.5 µg/mL.

Our results closely align with findings from the Mediterranean region, specifically Tunisia, where the antioxidant activity of *E. camaldulensis* on DPPH yielded IC_50_ values of 342 ± 2.5 µg/mL [24].

Conversely, in a separate study conducted in Thailand, the same DPPH assay was employed to assess the antioxidant potential of *E*. *camaldulensis* leaf essential oils using various solvents. The results from Thailand revealed higher IC_50_ values falling within the 1.75–12.62 mg/mL range. This disparity in results could potentially be attributed to variations in environmental factors such as climate and soil composition [25].

**Figure 2:**
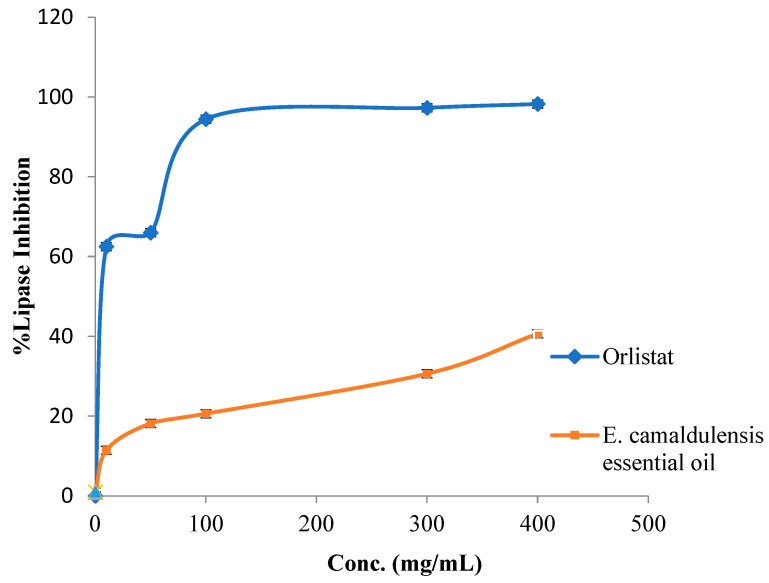
Porcine pancreatic lipase enzyme suppressant effect by *E. camaldulensis* essential oil and Orlistat.

Figure 2 shows that essential oil from *E. camaldulensis* was found to be inactive as an antilipase agent.

**Figure 3:**
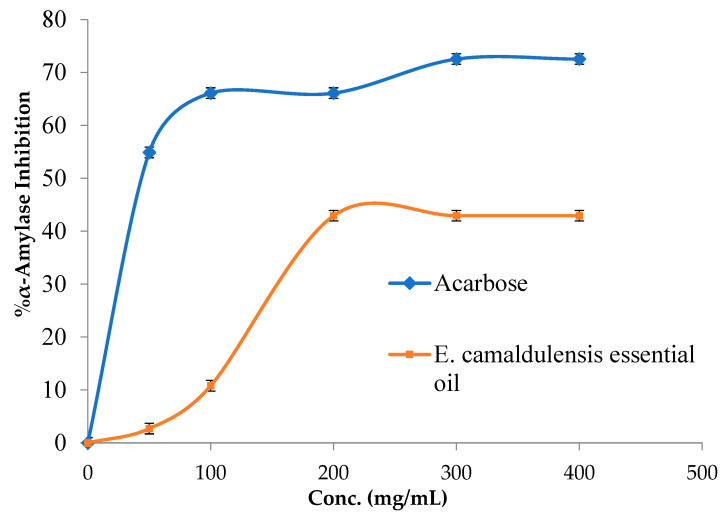
Porcine pancreatic α-amylase enzyme suppressant effect by *E. camaldulensis* essential oil and Acarbose.

The 3,5-dinitro salicylic acid (DNSA) method was employed in the present study to assess the *E. camaldulensis* essential oil antidiabetic effect by suppressing the effect of the α-amylase enzyme. Figure 3 shows that *E. camaldulensis* essential oil from Palestine was inactive against the α-amylase enzyme. A study conducted by Basak and Candan on the *E. camaldulensis* essential oil from Iran found that this oil has an anti-α-amylase effect with an IC_50_ value of 0.435 ± 0.003 μl/mL^−1^ [26]. This disparity can be attributed to variances in the chemical composition of the *E. camaldulensis* plant essential oil under investigation in our study, in contrast to the research conducted by Sahin Basak and Candan. Notably, the primary constituents identified in our study included *p*-cymene (38.64%), aromadendrene (29.65%), and 1,8-cineole (6.45%), whereas Sahin Basak and Candan reported dominant components in their research, namely *p*-cymene (68.43%), 1,8-cineole (13.92%), 1-(S)-α-pinene (3.45%), and R-(+)-limonene (2.84%).

### 2.3. Antimicrobial Effect

In our study, a microdilution assay was used to determine the antimicrobial effects of *E. camaldulensis* essential oil. Table 2 depicts that the *E. camaldulensis* essential oil has a broad-spectrum antimicrobial effect. The essential oil inhibited a strong effect against MRSA, *S. aureus*, and *P. vulgaris*, even more potent than the used positive controls Ciprofloxacin and Ampicillin antibiotics with MIC doses of 0.2 ± 0.01, 0.2 ± 0.01, and 6.25 ± 0.1 µg/mL, respectively. In addition, it showed a potent antifungal effect against *C. albicans* with a MIC of 0.2 ± 0.01 µg/mL compared with the Fluconazole antifungal drug (MIC = 1.56 ± 0.1 µg/mL). These results agreed with the previous investigation which found that *E. camaldulensis* essential oil had potential antimicrobial effects (Table 3).

High microbial resistance can be caused by many mechanisms, including multidrug resistance plasmids and biofilm formation [30,31]. Plants’ essential oils are considered a source of chemical structures that could inhibit microbial growth by employing many mechanisms [32]. Many studies have evaluated the relationship between the chemical structure of oxygenated compounds and the mechanisms of their antimicrobial action [33,34].

The abundant compound in our study is *p*-cymene [1-methyl-4-(1-methylethyl)-benzene] which is a monoterpene found in over 100 plant species utilized for medicine and food purposes. It showed a range of biological activity including, antimicrobial, anticancer, anxiolytic, antinociceptive, anti-inflammatory, and antioxidant effects. Previous studies have demonstrated that *p*-cymene affects liposomal membrane expansion and membrane potential. It also leads to changes in intracellular pH and ATP levels, which, in turn, suppress microbial growth [35].

In addition, a study conducted by Mulyaningsih et al. examined the antimicrobial activity of the fruit oil of *E. globulus* and the leaf oils of *E. globulus*, *E. radiata* Sieber ex DC, and *E. citriodora* Hook against multidrug-resistant bacteria. Furthermore, this study attempted to establish a relationship between the chemical composition and the corresponding antimicrobial properties. This study found the antimicrobial activity of aromadendrene, which is characterized as a major compound in plants, was stronger than that of 1,8-cineole against MRSA and Vancomycin-resistant *Enterococci* [36]. In fact, the aromadendrene compound has a reactive exocyclic methylene group and a cyclopropane ring which can alkylate proteins and, thereby, disturb the conformation of proteins. Additionally, since the compound is highly lipophilic, it can disrupt the fluidity and permeability of biomembranes [37,38].

1,8-cineole has exhibited antibacterial activity against different Gram-positive pathogenic strains, including *Staphylococcus epidermidis*, *Micrococcus flavus*, *Bacillus cereus*, *Streptococcus pyogenes*, *Bacillus subtilis*, and *S. aureus*, as well as Gram-negative strains, such as *Salmonella typhimurium*, *Proteus mirabilis*, *Enterobacter cloacae*, *Salmonella enteritidis*, *P. aeruginosa*, and *E. coli* [39].

Investigation outcomes by Li et al. demonstrated that 1,8-cineole can change the physical characters, including the shape and size of Gram-positive and Gram-negative bacteria [40].

Another study conducted by Hoch et al. found that 1,8-cineole had inhibitory effects on various fungi, including *Fusarium oxysporum*, *Fusarium sporotrichioides*, and *Aspergillus tubingensis* [41]. Therefore, the essential oil of *E. camaldulensis* from Palestine exhibited a strong antimicrobial effect, attributed to the presence of key molecules such as *p*-cymene, aromadendrene, and 1,8-cineole in the investigated plant.

## 3. Materials and Methods

### 3.1. Plant Material

The sample of the *E. camaldulensis* plant was identified by Dr. Nidal Jaradat in the Herbal Product Laboratory at An-Najah National University and deposited within a voucher specimen code (Pharm-PCT-2786). Then, the fresh leaves of *E. camaldulensis* were picked in June 2022 in the Jenin governorate of Palestine. The collected leaves were well washed with tap water and dried at a controlled temperature not exceeding 25 °C and a normal level of humidity without sunlight. The dried plant material was then powdered coarsely and stored in bags for further use.

### 3.2. Chemicals and Instruments

All of the chemical and biological components listed below were acquired from Frutarom (Clacton-on-Sea, UK): DPPH, Na_2_HPO_4_/NaH_2_PO_4_, NaCl, trolox, methanol, starch, porcine pancreatic amylase, lipase, 3,5-dinitro salicylic acid (DNSA), and Na_2_CO_3_. Dimethyl sulfoxide (DMSO) and Acarbose were obtained from Sigma-Aldrich, Steinheim, Germany. Several instruments were used in this study, including gas chromatography (PerkinElmer, Clarus 500, Waltham, MA, USA), mass spectrometer (Shelton, CT105484, Southlake, TX, USA), UV-visible spectrophotometer (Jenway 7135, Staffordshire, UK), a shaker device (Memmert shaking incubator, Buchenbach, Germany), a grinder (Moulinex, model LM2211, UNO, Shanghai, China), and a balance (Rad wag, AS 220/c/2, Radom, Poland).

### 3.3. Extraction of Essential Oil

Using a Clevenger apparatus, hydrodistillation was used to extract the *E. camaldulensis* EO. A round-bottom flask containing 500 g of plant material was filled with 1 L of distilled water, and the mixture was hydrodistilled for three hours. Before being used, the extracted EO was dried on anhydrous magnesium sulfate and kept in sealed, opaque glass vials at 4 °C. The yield in essential oil on a *w*/*w* basis was 1.11%.

### 3.4. Gas Chromatography-Mass Spectrometry

The phytochemical components of *E. camaldulensis* essential oil were qualitatively and quantitatively determined using gas chromatography (PerkinElmer, Clarus 500) linked with a Perkin Elmer Clarus 560 mass spectrometer (Shelton, CT105484). The separation was achieved by a Perkin Elmer Elite-5 fused-silica capillary column (film thickness 0.25 µm, 30 m × 0.25 mm). The temperature of the column was initially set at 50 °C and kept for 5 min. It was then set to rise at a rate of 4 °C per min until it reached a final temperature of 280 °C. Throughout all of the chromatographic runs, the flow rate of helium, which was utilized as a carrier gas, was kept constant at 1 mL/min. In total, 0.2 µL of the extracted *E. camaldulensis* EO was injected into a split mode with a splitting ratio of 1:50 and at 250 °C. The essential oil components were identified by comparing retention indices to data in the scientific literature and by matching their mass spectra to those in the NIST mass spectral database or those of pure reference compounds [42].

### 3.5. Free Radical Scavenging Activity

Using a previously described approach, we determined the essential oil’s ability to scavenge 2,2-diphenyl-1-picrylhydrazyl (DPPH) radicals [43]. In this experiment, a stock solution was subjected to serial dilution to obtain various concentrations (5, 7, 10, 20, 30, 50, 80, and 100 µg/mL). Subsequently, 1 mL of each concentration was combined with 1 mL of methanol and 1 mL of a 0.002% DPPH solution. The resulting mixtures were then incubated in darkness at room temperature for 30 min. Following incubation, the absorbance of the samples was measured at a wavelength of 517 nm. A range of Trolox stock solutions in methanol, with concentrations ranging from 10 mg/100 mL methanol, were prepared. Subsequently, 1 mL of each concentration was combined with 1 mL of methanol and 1 mL of 0.002% DPPH in a ratio of 1:1 (*v*/*v*). Following this, all samples were incubated in darkness at room temperature for 30 min. The absorption of each sample was then measured at a wavelength of 517 nm. A solution devoid of any substance was generated by combining equal volumes of DPPH solution and methanol. The absorbance of this solution was measured to facilitate the computation of the inhibition percentage.

% of inhibition = (B − Z)/B × 100%

B = absorbance of blank
Z = absorbance of the tested samples

### 3.6. Porcine Pancreatic Lipase Inhibition Assay

The porcine pancreatic lipase inhibition assay was utilized in this work, with modifications made based on a previous study [44]. A stock solution of plant essential oil at 1 mg/mL in 10% DMSO was used to prepare five distinct solutions with concentrations of 10, 50, 100, 300, 400 μg/mL by means of dilution with methanol. Just before usage, the pancreatic lipase enzyme was freshly produced as a 1 mg/mL stock solution. Additionally, a stock solution of PNPB (p-nitrophenyl butyrate) was created by dissolving 20.9 mg of PNPB in 2 mL of acetonitrile. In individual test tubes, 0.1 mL of porcine pancreatic lipase (1 mg/mL) was mixed with 0.2 mL of the essential oil concentrations (10, 50, 100, 300, and 400 μg/mL). To achieve a final volume of 1 mL, the Tri-HCl solution (pH 7.4) was added and the mixtures were incubated at 25 °C for 15 min. Following the incubation, 0.1 mL of PNPB solution was introduced to each test tube. The mixtures were once again incubated for 30 min at 37 °C. The assessment of pancreatic lipase activity involved measuring the hydrolysis of p-nitrophenyl butyrate to p-nitrophenol at 405 nm using a UV-visible spectrophotometer. The same procedure was replicated for Orlistat, which served as a positive control, with identical concentrations as mentioned earlier. All tests were conducted in triplicate.

% lipase inhibition = (AB − AE)/AB × 100%

where AB is the recorded absorbance of the blank solution and AE is the recorded absorbance of the oil sample solution.

### 3.7. α-Amylase Inhibitory Method 

The EO derived from the plant was initially solubilized in a minimal volume of dimethyl sulfoxide (DMSO) and subsequently diluted in a buffer solution (Na₂HPO₄/NaH₂PO₄ at 0.02 M, NaCl at 0.006 M, and adjusted to pH 6.9) to attain a concentration of 1 mg/mL. Various dilutions were subsequently prepared, including 50, 100, 200, 300, and 400 μg/mL. The reaction was initiated by combining 200 mL of a solution containing the porcine pancreatic α-amylase enzyme (2 units/mL) with 200 mL of plant essential oil. The mixture was then incubated at a temperature of 30 °C for a duration of 10 min. After the incubation period, 200 mL of freshly prepared starch solution (1% in water, *w*/*v*) was introduced into each tube and incubated for 3 min. The reaction was quenched by introducing 200 µL of DNSA reagent, which was prepared by mixing 12 g of sodium potassium tartrate tetrahydrate with 8.0 mL of 2 M NaOH and 20 mL of a 96 mM solution of 3,5-dinitrosalicylic acid. Subsequently, the solution was diluted using 5 mL of distilled water and subsequently subjected to heating for 10 min within a water bath that was consistently maintained at a temperature range of 85–90 °C. The mixture was then cooled to room temperature, and the absorbance at 540 nm was measured with a UV-visible spectrophotometer. To create a control with 100% enzyme activity, the plant essential oil was replaced with 200 mL of buffer. Additionally, a blank reaction was prepared for each concentration of the plant essential oil, devoid of the enzyme solution. A positive control sample, using acarbose, followed the same procedure as the one involving the plant essential oil. The α-amylase inhibitory activity was quantified as a percentage of inhibition, computed using the following formula:

% of a-amylase inhibition = (B − S)/B × 100%

where:

B: is the absorbance of blank;
S: is the absorbance of a tested sample [45].

### 3.8. Antimicrobial Activity

The antimicrobial activity of *E. camaldulensis* essential oil was evaluated using a broth microdilution assay on one fungal strain, *Candida albicans* (American Type Culture Collection (ATCC) 90028) and six bacterial strains, five of which were ATCC: *Pseudomonas aeruginosa* (ATCC 9027), *Escherichia coli* (ATCC 25922), *Klebsiella pneumonia*, (ATCC 13883), *Proteus vulgaris* (ATCC 8427), and *Staphylococcus aureus* (ATCC 25923), in addition to a diagnostically confirmed Methicillin-resistant *Staphylococcus aureus* (MRSA). 

The essential oil of *E. camaldulensis* was diluted to a concentration of 200 µg/mL in 20% dimethyl sulfoxide (DMSO). The solution was diluted ten times using sterile Muller-Hinton (RPMI media for the *C. albicans* strain) in a 2-fold serial microdilution. In 96-well plates, the dilution procedures were carried out in sterile conditions. 

Micro-well 11 was devoid of essential oil and was used as a positive control for microbial development, whereas micro-well 12 contained *E. camaldulensis* essential oil but was microbe-free. This well was utilized as a negative microbiological growth control. The test microorganisms were injected aseptically into microwells 1–11. All inoculation plates were incubated at 35 degrees Celsius. The incubation duration was approximately 18–24 h for plates infected with the test bacterial strains and approximately 48 h for plates inoculated with *Candida albicans*. The least inhibitory concentration (MIC) of the studied *E. camaldulensis* essential oil was determined to be the lowest concentration of essential oil at which no apparent microbial growth occurred in that micro-well. Ciprofloxacin and ampicillin were used as antibacterial activity controls in our technique, whereas fluconazole was used as an antifungal activity control. The antibacterial activity of *E. camaldulensis* essential oil was tested in triplicate [46].

### 3.9. Statistical Analysis 

All the experiments were carried out in triplicates. The results are presented as means ± standard deviation (SD). The results were considered significant only when the *p*-values were less than 0.005. 

## 4. Conclusions

This study demonstrates strong antimicrobial activity and weak antioxidant activity of essential oil from *E. camaldulensis*. No antiobesity or antidiabetic effect was observed with this essential oil in porcine pancreatic lipase and α-amylase inhibitory activity assays. All experimental findings demonstrate that *E. camaldulensis* essential oil is rich in phytochemicals but has weak antioxidant activity compared with Trolox while it has no antidiabetic and antiobesity properties. Also, *E. camaldulensis* essential oil showed a strong antimicrobial effect, especially against *C. albicans*, MRSA, *S. aureus*, and *P. vulgaris*. In light of these findings, advanced in vivo studies are imperative to substantiate the antimicrobial efficacy of *E. camaldulensis* essential oil on living organisms and to formulate appropriate pharmaceutical dosage forms for the treatment of infectious diseases.

## Figures and Tables

**Table 1:** Phytochemical composition of *E. camaldulensis* essential oil collected from Palestine.

Compounds	Retention Time (min)	Kovats’ Retention Index *	Area	Relative Area (%)
α-Thujene	9.471	923	1,599,865	0.57
α-Pinene	9.791	931	2,615,981	0.94
Thuja-2,4(10)-diene	10.246	941	480,772	0.17
Sabinene	11.541	970	139,685	0.05
β-Pinene	11.741	974	86,508	0.03
Myrcene	12.337	987	235,294	0.09
α-Phellandrene	13.022	1002	733,936	0.27
α-Terpinene	13.497	1014	628,687	0.23
*p*-Cymene	13.867	1022	106,797,504	38.64
Sylvestrene	14.067	1027	6,566,526	2.38
1,8-Cineole	14.152	1029	17,823,158	6.45
γ-Terpinene	15.298	1056	936,553	0.34
Terpinolene	16.433	1083	152,116	0.06
*p*-Cymenene	16.613	1087	929,263	0.34
*cis*-Thujone	17.264	1102	835,748	0.30
cis-p-Menth-2-en-1-ol	17.719	1114	549,290	0.20
α-Campholenal	18.079	1123	113,198	0.04
Allo-ocimene	18.394	1131	533,340	0.19
4-Ketoisophorone	18.574	1136	318,614	0.12
Sabina ketone	18.984	1147	1,808,566	0.65
Borneol	19.77	1167	526,830	0.19
4-Ethyl-3,4-dimethyl-2-cyclohexene	19.92	1171	402,158	0.15
Terpinen-4-ol	20.25	1179	8,409,609	3.04
Verbenone	20.8	1193	417,699	0.15
γ-Terpineol	21	1198	1,443,225	0.52
α-Terpineol	21.23	1204	248,590	0.09
Citronellol	21.885	1223	114,947	0.04
Ascaridole	22.361	1236	224,751	0.08
Cuminaldehyde	22.486	1239	14,441,493	5.22
Ethyl oct-(2E)-enoate	22.781	1248	381,282	0.14
Piperitone	22.896	1251	646,349	0.23
n-Decanol	23.291	1262	237,868	0.09
Phellandral	23.756	1279	11,838,921	4.28
α-Terpinen-7-al	24.056	1283	427,241	0.15
Carvacrol	24.862	1306	641,374	0.23
Myrtenyl acetate	25.347	1321	313,433	0.11
3-oxo-ρ-Menth-1-en-7-al	25.807	1334	392,091	0.14
Bicyclo [3,3,1]non-6-en-3-ol, 7-methyl	26.377	1351	142,510	0.05
α-Ylangene	27.132	1374	64,722	0.02
n-Tetradecane	27.908	1397	144,223	0.05
α-Santalene	28.493	1416	119,993	0.04
α-Humulene	29.633	1452	201,051	0.07
Allo-aromadendrene	29.849	1459	2,210,432	0.80
n-Pentadecane	31.039	1497	159,306	0.06
14-Hydroxy-9-epi-(E)-caryophyllene	35.751	1660	1,375,888	0.50
Isobicyclogermacrenal	37.732	1733	567,702	0.21
Cyclocolorenone	38.322	1755	477,660	0.17
Methyl octanoate	17.564	1110	129,609	0.05
β-Santalene	30.084	1467	489,471	0.18
α-Copaene	30.734	1488	1,912,648	0.69
Aromadendrene	33.47	1598	81,966,904	29.65
epi-α-Cadinol	34.455	1614	1,450,303	0.57
**Total identified**			276,404,887	100.00
**Phytochemical classification**				
Monocyclic monoterpene				44.27
Oxygenated monoterpenoid				21.62
Sesquiterpenes hydrocarbon				31.46
Oxygenated sesquiterpenoid				1.44
Others				1.21
**Total**				100.00

* Retrieved from National Institute of Standards and Technology, U.S. Department of Commerce, NIST Chemistry WebBook, SRD 69 (https://webbook.nist.gov/chemistry/name-ser/ (accessed on 6 July 2023)).

**Table 2:** Antimicrobial effects MIC (µg/mL) of *E. camaldulensis* essential oil and positive controls.

	Bacteria						Fungus
	Gram-Positive		Gram-Negative				Yeast
ATCC Number/Strain	Clinical Strain	ATCC 25923	ATCC 25922	ATCC 13883	ATCC 8427	ATCC 9027	ATCC 90028
Microbe	MRSA	*S. aureus*	*E. coli*	*K. pneumoniae*	*P. vulgaris*	*P. aeruginosa*	*C. albicans*
***E. camaldulensis* essential oil**	0.2 ± 0.01	0.2 ± 0.01	12.5 ± 0.09	12.5 ± 0.09	6.25 ± 0.1	50 ± 1.19	0.2 ± 0.01
**Ciprofloxacin**	12.5 ± 0.09	0.78 ± 0.01	1.56 ± 0.1	0.13 ± 0.01	15 ± 1.1	3.12 ± 0.35	R
**Ampicillin**	25 ± 0.1	25 ± 0.125	3.12 ± 0.35	1.25 ± 0.1	18 ± 1.04	R	R
**Fluconazole**	R	R	R	R	R	R	1.56 ± 0.1

Where R: Resistance.

**Table 3:** Antimicrobial effects of *E. camaldulensis* essential oil from different studies.

Microbial Name	MIC Values	Reference
MRSA (2)	1000 µg/mL	[27]
*S. aureus*	50 mg/mL	[28]
*K. pneumoniae*	25 mg/mL	
*P. mirabilis*	25 mg/mL	
*E. coli*	50 mg/mL	
*P. aeruginosa*	4 mg/ mL	[5]
*C. albicans*	150 µg/mL	[29]

## Data Availability

All data is contained within the article.
